# Novel Green Strategy to Recover Bioactive Compounds with Different Polarities from Horned Melon Peel

**DOI:** 10.3390/foods13182880

**Published:** 2024-09-11

**Authors:** Teodora Cvanić, Mirjana Sulejmanović, Milica Perović, Jelena Vulić, Lato Pezo, Gordana Ćetković, Vanja Travičić

**Affiliations:** 1 Faculty of Technology Novi Sad, University of Novi Sad, Bulevar cara Lazara 1, 21000 Novi Sad, Serbia; teodora.cvanic@uns.ac.rs (T.C.); mirjana.sulejmanovic@uns.ac.rs (M.S.); perovicmilica@uns.ac.rs (M.P.); jvulic@uns.ac.rs (J.V.); gcetkovic@uns.ac.rs (G.Ć.); 2 Engineering Department, Institute of General and Physical Chemistry, Studentski trg 12/V, 11000 Belgrade, Serbia; latopezo@yahoo.co.uk

**Keywords:** horned melon peel, polyphenols, carotenoids, cloud point extraction, green chemistry, antioxidant activity, OVAT, Plackett–Burman design, ANN

## Abstract

Around 20–30% of the horned melon’s weight is peel. This peel is often discarded or underutilized despite containing valuable bioactive compounds. Conventional methods for extracting polyphenols and carotenoids from horned melon peel are typically inefficient, environmentally harmful, or require significant time and energy. The potential of green cloud point extraction (CPE) or green surfactant-based extraction for recovering bioactives with different polarities from this kind of by-product has not been thoroughly investigated. Therefore, this study focused on optimizing CPE process parameters using a one-variable-at-a-time (OVAT) approach. Optimal CPE demonstrated superior yields compared to conventional, ultrasound, microwave, ultrasound-assisted CPE, and microwave-assisted CPE methods. Further, a Plackett–Burman design identified key factors influencing optimal CPE conditions, while artificial neural network (ANN) analysis assessed each input variable’s impact on outcomes. Maximum extraction efficiency for total phenolics (352.49 mg GAE/100 g), total carotenoids (16.59 mg β-carotene/100 g), and antioxidant activity (989.02 μmol TE/100 g) was achieved under conditions of: surfactant type = Tween 80, surfactant concentration = 2%; solid:liquid ratio = 1:100; pH = 6612; equilibration temperature = 35 °C; equilibration time = 60 min; salt type = NaCl; salt concentration = 16.4%; centrifugation speed = 7906× *g* ; centrifugation time = 13.358 min; and No. of CPE steps = Step 1. This comprehensive approach aimed to enhance the understanding and optimization of CPE for maximizing the recovery of bioactives from the horned melon peel, addressing the inefficiencies of traditional extraction methods.

## 1. Introduction

Bioactive compounds such as phenolics, carotenoids, and vitamins are vital for human health and are widely used in pharmaceuticals and nutraceuticals due to their antioxidant properties. The increasing interest in these bioactives is pushing the development of new extraction processes from various natural sources [1]. Conventional extraction methods often use toxic chemicals that cause environmental threats and have a long extraction time, followed by partial recovery and degradation of bioactive compounds [2]. In contrast, novel eco-friendly technologies, including supercritical fluid extraction, ultrasound-assisted extraction, and microwave-assisted extraction, provide maximum recovery in shorter times using green solvents, minimizing environmental impact [2,3]. These methods align with green chemistry principles and are promising alternatives to conventional techniques.

Among various green extraction methods, cloud point extraction (CPE) is recognized as a modern and highly environmentally sustainable, cost-effective technique for the separation and preconcentration of bioactive compounds [4]. It is also known as a liquid-concentration technique, or micelle, that uses surfactant to extract organic or inorganic parts in separation science. The extraction occurs at a cloud point temperature where the surfactant becomes cloudy (less soluble or even insoluble), resulting in the separation of two phases: the surfactant-rich phase and the aqueous phase, where mainly bioactive compounds concentrate within the surfactant-rich phase [4,5]. CPE is one of the promising and bright future techniques that can be applied for the extraction of bioactives from food processing raw materials or food waste, as well as the separation and purification of different functional components.

In recent times, consumers’ needs for exotic fruits have increased worldwide, mainly due to their knowledge regarding their bioactive composition and the numerous health benefits. Horned melon, or kiwano (*Cucumis metuliferus* E. Mey. Ex. Naudin), is fruit that is rich in nutritional and phytochemical components and widely cultivated and consumed for its sweet, succulent flesh and high water content [6]. Consuming kiwano is linked to several health advantages, such as antifungal, antibacterial, antiviral, antihypertensive, antidiabetic, and antioxidant properties. Evaluation of the phytochemical analysis revealed the presence of carotenoids, steroids, alkaloids, saponins, glycosides, flavonoids, tannins, and phenolic compounds [7]. The ripe pulp of the fruits is consumed raw in its fresh form and can equally be processed into fruit juice [8]. Increased productivity of this fruit is accompanied by the generation of huge amounts of horned melon non-edible parts (e.g., peels and seeds) that are considered renewable sources of added-value bioactive compounds. The valorization of these by-products offers a sustainable waste management solution with great potential for the food industry.

To the best of our knowledge, there are no studies concerning the extraction of polyphenols and carotenoids from horned melon peel using CPE. In a recent review article, Travičić et al. [9] described innovative strategies that integrate cutting-edge technologies such as ultrasound or microwave assistance with CPE. The extraction of multiple bioactive compounds simultaneously holds immense potential for scientific advancements and practical applications. In response to the increasing interest among researchers in developing novel extraction methods and filling the current knowledge gap, this study investigated the potential of CPE for the extraction of bioactive compounds with different polarities from horned melon peel as a waste matrix. These extracts could potentially be utilized in the formulation of food and nutraceutical products as eco-friendly alternatives.

## 2. Materials and Methods

### 2.1. Plant Material and Other Chemicals

Horned melon fruits were purchased from an organic agricultural holding at the Fruška gora Mountain, in the area of Pannonia Plain, Novi Sad, Republic of Serbia (45°12′ N, 19°45′ E). The horned melon peel was separated manually in aseptic laboratory conditions and stored after being freeze-dried. Briefly, the main drying process was performed in a Martin Crist Alpha 2–4 (Osterode, Germany) freeze-drier at a pressure of 0.01 mbar and temperatures from −40 to 20 °C for 48 h. The final drying lasted 4 h at pressure of 0.005 mbar and temperatures from 20 to 30 °C. The collected freeze-dried horned melon peel was powdered using a laboratory grinder (B800E high-speed grinder, Gorenje, Velenje, Slovenia), and 71.5 µm was the particle size of plant material determined on a sieve set (CISA Cedaceria Industrial, Spain). The samples were stored at freezing temperature prior to further use. The dry matter content for horned melon peel measured after drying at 105 °C for 24 h was 13.16%.

Triton X-100 and Span 85 were purchased from Sigma Chemical Co. (St. Louis, MO, USA). Tween 80 was obtained from Alfa Aesar (Karlsruhe, Germany), while Ceteareth 12 (Brij CS12) was purchased from Avena Lab—Farmadria d.o.o. (Vršac, Serbia). All other chemicals and solvents used for assays were of the highest analytical grade.

### 2.2. One Factor at a Time Design (OVAT)

CPE was conducted using a modified method of Motikar et al. [10]. The one-factor-at-a-time (OVAT) approach was used to determine the optimum extraction conditions. Horned melon peel powder, water, and different surfactants were added in 100 mL conical flask with different solid-to-liquid ratio (*w*/*v*), surfactant concentration (%, *w*/*v*), and pH of solution, respectively. The mixture was thoroughly mixed for 20 min on a magnetic stirrer, followed by centrifugation at 2473× *g* for 10 min and separation of supernatant. The supernatant was mixed with the salt (NaCl) at different concentrations (%, *w*/*v*). Further, the mixture was kept in the water bath at varying temperatures and time, followed by centrifugation to accomplish phase separation. Table 1 provides an illustration of the variable extraction conditions. The bottom water phase was separated by pipette, and the remaining surfactant phase was used for estimation of total polyphenols (TPh), total carotenoids (TCar), and antioxidant activity (AA).

### 2.3. Conventional Water Extraction

Horned melon peel powder (1 g) was extracted in 50 mL of distilled water for 3 h on a magnetic stirrer at 40 °C. The liquid extract was separated by centrifugation at 2473× *g* for 10 min. The supernatant was analyzed for TPC, TFC, and AA.

### 2.4. Ultrasound Extraction and Ultrasound-Assisted CPE

The ultrasound extraction of bioactive compounds (polyphenols and carotenoids) from horned melon peel powder was performed in a sonication water bath (Grant Instruments, Royston, UK). Horned melon peel powder and water mixture in 1:50 ratio was extracted at a temperature of 60 °C and frequency of 40 kHz with constant power of 140 W for 30 min. After the extraction, the sample solution was centrifuged at 2473× *g* for 10 min, and the supernatant was collected.

For ultrasound-assisted CPE, ultrasound bath was set at 60 °C for 30 min, frequency at 40 kHz, and power at 140 W. The CPE steps were repeated using optimized CPE parameters.

### 2.5. Microwave Extraction and Microwave-Assisted CPE

Microwave extraction was conducted by mixing 2.5 g of powdered horned melon peel in 50 mL of distilled water at a microwave power of 600 W for 60 s. The liquid extract was collected by separating solids by centrifugation at 2473× *g* for 10 min.

For microwave-assisted CPE, horned melon peel powder and distilled water in 1:50 ratio and Tween 80 (10% *v*/*v*) were mixed and kept in the microwave at 600 W for 60 s. Then, the CPE steps were repeated using optimized CPE parameters.

### 2.6. Analytical Methods

#### 2.6.1. Determination of Total Polyphenols (TPC)

Total polyphenol content (TPC) of the extracts was established using the Folin–Ciocalteau spectrophotometric method adapted to microscale [11]. Each microplate well contained a mixture of 15 μL of sample, 170 μL of distilled water, 12 μL of the Folin–Ciocalteu’s reagent, and 30 μL of 20% (*w*/*v*) sodium carbonate. The prepared microplate was incubated for 1 h, and the absorbances were measured at 750 nm. Distilled water was used as blank. The obtained results were expressed as mg gallic acid equivalents (GAE) per 100 g of sample dry weight (dw).

#### 2.6.2. Determination of Total Carotenoids (TC)

Total carotenoid content (TC) in the extracts was determined spectrophotometrically as described by Šeregelj et al. [12]. Extracting solvent was used as a blank. The total carotenoid content was expressed as mg of β-carotene equivalents per 100 g of sample dry weight (dw).

#### 2.6.3. Determination of Antioxidant Activity (AA)

The DPPH radical scavenging assay was performed spectrophotometrically according to Tumbas Šaponjac et al. [12]. In brief, 250 μL of DPPH^•^ solution in methanol (0.89 mM) was mixed with 10 μL of sample in a microplate well. Absorbance was measured at 515 nm after 50 min of incubation in the dark at ambient temperature. Methanol was used as a blank. The results were expressed in μmol Trolox equivalent (TE) per 100 g of sample dry weight (dw).

### 2.7. HPLC Analysis of Horned Melon Peel Extracts

#### 2.7.1. HPLC Phenols Profiling

Horned melon peel extracts were analysed by a chromatographic system Shimadzu Prominence (Shimadzu, Kyoto, Japan). Chromatograms were recorded using different wavelengths for individual compounds: 280 and 320 nm for phenolic acids and 360 nm for flavonoids. Separation was performed on a Luna C-18 RP column, 5 mm, 250 mm × 4.6 mm with a C18 guard column, 4 mm × 30 mm (both from Phenomenex, Torrance, CA, USA) and analyzed by Diode Array Detector SPD-M20A (Shimadzu, Kyoto, Japan). Two mobile phases, A (acetonitrile) and B (1% formic acid), were used at a flow rate of 1 mL/min with the following gradient profile: 0–10 min from 10 to 25% B, 10–20 min linear rise up to 60% B and from 20 to 30 min linear rise up to 70% B, followed by 10 min reverse to initial 10% B with additional 5 min of equilibration time. The results are reported as mg/100 g sample dry weight (dw).

#### 2.7.2. HPLC Carotenoids Profiling

Carotenoids profiling was performed by reverse-phase HPLC. Briefly, 5 mL of horned melon peel extract was weighted in a 25 mL screw-capped tube, wrapped in aluminum foil, and saponified under nitrogen for 45 min at 70 °C in thermostatic bath (Haake E8, Berlin, Germany), with the addition of 2.5 mL of ethanolic pyrogallol (60 g/L) as antioxidant, 1 mL of ethanol (95%), 1 mL of sodium chloride (10 g/L), and 1 mL of potassium hydroxide (600 g/L). During the saponification, the tubes were vortexed every 10 min. Afterwards, they were cooled in an ice bath, and 7.5 mL of sodium chloride (10 g/L) were added. The suspension was then extracted with 15 mL of hexane:ethyl acetate (9:1 *v*/*v*). The organic layer was collected and evaporated under vacuum, followed by nitrogen drying; the residue was dissolved in 1 mL methanol: tetrahydrofuran (95:5 *v*/*v*) and filtered through a 0.45 μm PTFE membrane. The filtered solution was analysed by RP-HPLC following Šeregelj et al. [13] using a column PerkinElmer Quasar C18, 250 × 4.6 mm, 5 μm (PerkinElmer, Buckinghamshire, UK); mobile phase, methanol:tetrahydrofuran stabilized with 0.1% butylated hydroxytoluene (95:5, *v*/*v*); flow rate, 1 mL/min. Carotenoids were detected at 445 nm, using a Waters 996 series photodiode array detector SPD-M20A (Shimadzu, Kyoto, Japan). The wavelength range was 200–600 nm. The peaks of all components were detected at 445 nm. The carotenoid compounds were quantified based on the area under the detected peaks against calibrated (r^2^ > 0.99) standards. The results are expressed in mg/100 g of sample dry weight (dw).

### 2.8. Statistical Analysis

Statistical processing of the data was conducted using STATISTICA 10.0 software (StatSoft Inc., Tulsa, OK, USA). Each analysis was performed in triplicate. Prior to analysis, all samples underwent assessments for variance equality (Levene’s test) and normal distribution (Shapiro–Wilk’s test). Variations in parameters among samples were explored through analysis of variance, supplemented by Tukey’s HSD post hoc test for comparing sample means.

#### 2.8.1. Artificial Neural Network (ANN) Modelling

A laboratory-scale model was implemented to apply the Plackett–Burman’s screening design (Table 2), aiming to investigate the primary effects of 11 process variables (surfactant type—X1; surfactant concentration—X2; solid:liquid ratio—X3; pH—X4, equilibration temperature—X5; equilibration time—X6; salt type—X7; salt concentration—X8; centrifugation speed—X9; centrifugation time—X10; and the number of CPE steps—X11). This experimental setup facilitated the assessment of variable importance and the identification of key factors for further enhancement. An artificial neural network (ANN) model, specifically a multi-layer perceptron (MLP) comprising input, hidden, and output layers, was constructed to predict the optimal CPE extraction parameters based on the 11 variables stipulated by the Plackett–Burman experimental design. To enhance ANN performance, the database underwent normalization before calculations were executed. Various ANN topologies were explored during training by building 100,000 different ANNs with randomized initial values for weights and biases, and with various numbers of hidden neurons ranging from 5 to 20. The Broyden–Fletcher–Goldfarb–Shanno (BFGS) algorithm served as the iterative method for addressing unconstrained nonlinear optimization challenges encountered during ANN optimization [14].

#### 2.8.2. Global Sensitivity Analysis

Yoon’s global sensitivity equation was employed to quantify the influence of input parameters on output variables, based on the weight coefficients derived from the ANN models as outlined by Yoon et al. [15].

## 3. Results and Discussion

### 3.1. OVAT Approach

#### 3.1.1. Selection of Surfactant for CPE

The crucial characteristic of surfactants is their solubility, which stems from having both hydrophilic and hydrophobic parts. This underscores the importance of selecting an appropriate surfactant for a particular system to effectively collect and maximize the yield of both solvent–soluble and insoluble compounds within micelles [16]. In previous research, the extraction efficiency of polyphenols and carotenoids was explored using various surfactant types, including cationic, anionic, and nonionic. Researchers observed that employing nonionic surfactants resulted in a higher yield of target components [17,18]. Additionally, nonionic surfactants have lower critical micelle concentrations, enabling their use at lower concentrations. Based on these findings, this study focuses on various categories of nonionic surfactants, each with unique physicochemical properties.

The screening of four surfactants, i.e., Tween 80, Triton X-100, Ceteareth-12, and Span 85, was conducted, aiming to verify their efficiency for maximum extraction yield of target bioactives. The ongoing parameters included a solid-to-liquid ratio of 1:50, a concentration of surfactant of 4% (*w*/*v*) at 45 °C, pH 4, for 30 min, and a salt concentration of 15% (*w*/*v*), followed by centrifugation at 2473× *g* for 10 min. The obtained results are shown in Table 3.

Among the investigated surfactants, there was no phase separation observed with Ceteareth-12 and Span 85, whereas Triton X-100 and Tween 80 resulted in two distinct phases in the samples. It was found that the use of Tween 80 resulted in 81.72 mg GAE/100 g dw of TPC, 6.27 mg β-carotene/100 g dw, and an AA of 226.47 μmol TE/100 g dw. In contrast, employing Triton X-100 led to significantly lower concentrations of TPC (20.93 mg GAE/100 g dw), TC (5.81 mg β-carotene/100 g dw), and AA (140.14 μmol TE/100 g dw). These findings align with the research by Katsoyannos et al. [19]. They found that Tween 80 showed the highest recovery rates for phenolics (96.4%) and carotenoids (64.3%). Skrypnik and Novikova [18] also reported a higher efficiency of Tween 80 for polyphenol extraction from apple pomace in comparison to Triton X-100, Span 20, and Tween 20.

The observed differences in phase separation among the investigated surfactants can be attributed to their hydrophilic–lipophilic balance (HLB). Based on their HLB values, the surfactants ranged as follows: Span 85 (1.8), Ceteareth-12 (12), Triton X-100 (13.5), and Tween 80 (15). Tween 80 showed the highest ability to enhance the extraction process of polyphenols and carotenoids, which is in correlation with the HLB value (highest value). Based on the literature data, it can be observed that the efficiency of surfactant depends not only on its HLB properties, but also on the composition of compounds in plant material. For example, the use of Tween 80 for the extraction of polyphenols from rattan tea (*Ampelopsis grossedentata*) exhibited the highest efficiency [20], the Brij-58 [21] and Brij-35 [17] solutions were the most effective in the extraction of phenolic compounds from fruits, and the Triton X-100 solution was the most effective for the extraction of conjugated phenolic compounds from black tea [22]. Thus, selecting an appropriate surfactant for CPE requires considering the plant raw material matrix and the qualitative composition of the extracted compounds.

#### 3.1.2. Effect of Surfactant Concentration

After screening various surfactants, Tween 80 was chosen for further investigation. Quina and Hinze [16] noted that the extraction efficiency increases up to a maximum point with higher surfactant concentrations. However, according to More and Arya [23], it is recommended to keep concentration below 11%. Therefore, the concentration of surfactant was varied between 2 and 10% to select the most efficient surfactant concentration, and obtained results are presented in Table 4.

Increasing the surfactant concentration significantly enhanced the TPC and AA, while TC remained relatively unchanged for surfactant concentration above 4%. Specifically, at a 10% surfactant concentration, the TPC was 150.97 mg GAE/100 g dw, compared to 62.59 mg GAE/100 g dw at a 2% concentration, demonstrating the substantial impact of higher surfactant levels. A similar trend was observed for AA, with DPPH assay results showing values of 204.85 and 409.14 μmol TE/100 g dw at 2% and 10% surfactant concentrations, respectively. The TC was approximately 10 mg β-carotene/100 g dw for surfactant concentrations above 4%. These findings are consistent with Kiai et al. [24], who reported maximum polyphenolic recovery from table olive processing wastewaters with a 10% Tween 80, as well as with Katsoyannos et al. [19], who found that 10% Tween 80 yielded the highest recovery of phenolics and carotenoids from red-flesh orange juice and olive mill wastewater.

#### 3.1.3. Effect of Solid-to-Liquid Ratio

Optimizing the solid-to-liquid ratio is crucial for efficient extraction of bioactive compounds, balancing resource use and extraction completeness. Using higher than optimal solvent levels can result in waste while using lower levels can lead to incomplete extraction of bioactives [10]. Table 5 shows the effect of the solid-to-liquid ratio on target responses. CPE was performed at varied solid-to-liquid ratios ranging from 1:10 to 1:100 with 10% *w*/*v* Tween 80.

Table 5 reveals that all responses (TPC, TC, and AA) gradually increased as the ratio of solid-to-liquid increased from 1:10 to 1:50. However, a slight decrease was noted at ratios of 1:70 and 1:100. It can be explained due to the fact that solute concentration was less, leading to decreased extraction yield. The most favourable solid-to-liquid ratio was 1:50, where TPC, TC, and AA were 201.35 mg GAE/100 g dw, 10.75 mg β-carotene/100 g dw, and 617.73 μmol TE/100 g dw, respectively. A similar observation was recorded for the CPE extraction of pomegranate peel by Živkovic et al. [25].

#### 3.1.4. Effect of pH

The extraction yields of most substances vary with changes in pH. According to the literature data, in terms of polyphenols, the optimal pH values are located in the acidic region [4,17,26]. Depending on the acidity, phenolic compounds can exist in either neutral or ionic forms. Simple phenolic compounds are typically weak acids with pKa values ranging from 2.5 to 4.9. At acidic pH, phenolic compounds predominantly remain in their undissociated state, making them more likely to interact with the hydrophobic micellar phase. Conversely, pH values above 8 can cause the dissociation of bioactive compounds, leading to decreased recovery of these substances [23]. The effect of pH on CPE yield was examined by varying the pH from 1.5 to 7.5, adjusted using 0.1 N HCl or 0.1 N NaOH (Table 6), with a 10% *w*/*v* Tween 80 solution at a 1:50 solid-liquid ratio.

The highest TPC and AA were observed for pH 3, reaching 198.86 mg GAE/100 g dw and 622.42 μmol TE/100 g dw, respectively. However, in a sample where pH was 3, the TC (5.50 mg β-carotene/100 g dw) was lower compared to higher pH values. Giovanoudis et al. [27] found that the maximum recovery of carotenoids from liquid tomato wastewater occurred at pH 3.5. Alibade et al. [4] reported that using lecithin as a surfactant resulted in maximal recovery at a pH of 3. Similarly, Stamatopoulos et al. [28] discovered that the optimal pH for implementing Tween 80 was 3 for olive leaf extraction. The observed variability in carotenoid yield at different pH levels indicates the strong relationship between pH variations, surfactants, and the nature of the matrix in extraction processes. Carotenoids exhibit a notable sensitivity to pH changes, impacting their stability, solubility, and interactions with surrounding compounds [29]. Lower pH levels may trigger carotenoid degradation or complex formation. Additionally, the specific matrix or source material significantly influences this process, introducing various interfering substances that alter carotenoid behavior under different pH conditions. This complex interaction underscores the need for ongoing research and optimization to fully understand and control these factors to maximize carotenoid recovery.

#### 3.1.5. Effect of Equilibration Temperature

The temperature at which a surfactant’s solubility in water significantly increases is known as the Krafft point, and it is generally regarded as the melting point of a hydrated solid surfactant. Beyond the Krafft point, the surfactant’s overall solubility rises markedly due to micelle formation, facilitating effective extraction [30]. Additionally, the equilibration temperature is influenced by salt concentration, which dehydrates the polyoxyethylene chain of non-ionic surfactants. Consequently, as salt concentration increases, the cloud point temperature (CPT) decreases [24]. Optimizing the equilibration temperature is crucial, as phase separation will not occur if the equilibration temperature is below the CPT. Therefore, various temperatures between 35 and 75 °C were examined to determine the suitable equilibration temperature for this system (Table 7).

Table 7 reveals that as the temperature increased from 35 °C to 45 °C, there was a gradual rise in all measured responses. The highest yields recorded were 279.81 mg GAE/100 g dw for TPC, 2.70 mg β-carotene/100 g dw for TC, and 1085.39 μmol TE/100 g dw for AA. However, beyond 45 °C, the yields of these bioactives and the antioxidant activity began to decline, likely due to the degradation of polyphenols and carotenoids at temperatures higher than 45 °C. This phenomenon is consistent with findings from Giovanoudis et al. [31], who also used an equilibration temperature of 45 °C in their study on the extraction of ripe and unripe peaches by CPE. Additionally, Stamatopoulos et al. [28] examined the effect of temperature on phase separation when 4% Tween 80 and 35% salt were used. Furthermore, Giovanoudis et al. [27] investigated the impact of temperature (25–65 °C) on carotenoid extraction from liquid tomato wastewater, concluding that the optimal temperature was 45 °C.

#### 3.1.6. Effect of Equilibration Time

The equilibration time is an important parameter as it dictates the duration of contact between bioactive compounds and the surfactant that leads to the formation of micelles. The evaluation of equilibration time provides information regarding sufficient contact time for maximal recovery of bioactive compounds [24]. Therefore, this study evaluated five different equilibration times (20, 30, 40, 50, and 60), and the results are shown in Table 8.

The investigation into equilibration times revealed minimal differences between 20 and 30 min for TPC and AA. The TC was notably higher at 20 min (3.29 mg β-carotene/100 g dw), compared to longer durations of 30, 40, 50, and 60 min, where lower carotenoid levels were observed. Additionally, at 40, 50, and 60 min, TPC dropped below 194.62 mg GAE/100 g dw. The 20 min equilibration time was also employed in other studies by Giovanoudis et al. [31,32], examining various fruits. These findings suggest that shorter equilibration times positively affect the recovery of target compounds, leading to the selection of 20 min as the optimal parameter for the CPE procedure.

#### 3.1.7. Effect of Salt Type

The salt addition increases both the quantity and size of micelles. Salt primarily functions to lower the cloud point temperature, thereby safeguarding heat-sensitive substances and intensifying the hydrophobic interactions between surfactants and analytes [33]. Commonly used neutral salts like NaCl, KCl, and CaCl_2_ are typically employed for this purpose. According to Chawla and Mahajan [34], the cloud point temperature of Tween solutions decreases when NaCl and KCl are introduced, as they disrupt the hydration layers surrounding the polyoxyethylene head groups of Tween molecules through a salting-out effect.

However, in this study, phase separation was only observed with NaCl (Table 9). The TPC, TC, and AA were 295.92 ± 2.67 mg GAE/100 g dw, 3.59 ± 0.74 mg β-carotene/100 g dw, and 1095.84 ± 10.97 μmol TE/100 g dw, respectively. NaCl usage can be seen in the study by Giovanoudis et al. [31], where Tween 80 was also employed as a surfactant.

#### 3.1.8. Effect of Salt Concentration

Apart from selecting the most suitable type of salt for the system under study, the concentration also significantly influences phase separation. This phenomenon may be linked to the salting-out effect, where the surfactant phase experiences dehydration, thereby facilitating effective phase separation [23].

In experiments using 10% and 14% salt concentrations, no phase separation occurred, indicating homogenous solutions (Table 10). However, higher salt concentrations resulted in the formation of two distinct phases. Specifically, at 18% salt concentration, the surfactant-rich phase exhibited increased viscosity. This phase behavior adversely affected the volumetric ratio and subsequently led to reduced yields of bioactive compounds [35]. Higher values for all three responses were achieved with 16% NaCl, suggesting an optimal salt concentration for maximizing the extraction efficiency of target bioactive compounds.

#### 3.1.9. Effect of Centrifugation Speed

According to Xu et al. [36] centrifugation speeds typically exert a modest influence on micelle formation, consequently showing minimal impact on CPE efficiency. Nevertheless, the effect of centrifugation speed was investigated within the range of 2473 to 9892× *g* (Table 11).

Lower centrifugation speed (2473× *g*) led to increased recovery of bioactive compounds: 296.67 mg GAE/100 g dw for TPC, 7.02 mg β-carotene/100 g dw for TC, and 740.32 μmol TE/100 g dw for AA. Xu et al. [36] varied centrifugation time from 5 to 20 min and centrifugation speed from 4000 to 6000 rpm. Their findings indicated that optimal values for targeted flavonoids were achieved with a centrifugation time of 10 min at 4000 rpm, aligning with the results in the present study.

#### 3.1.10. Effect of Centrifugation Time

According to Paleologos et al. [37], an effective centrifugation time for CPE ranges from 5 to 10 min. In this study, the effects of three different durations (10, 15, and 20 min) were assessed, and the findings are detailed in Table 12.

Centrifugation time of 10 min resulted in achieving the highest values across all tested outcomes. This finding correlates with the findings of Zafar et al. [38]. The observed lower recovery of target bioactives and antioxidant activity at longer centrifugation times (15 and 20 min) can be attributed to the increased force applied to the sample during these durations. While these longer times enhance the separation efficiency of components, they also risk compromising the stability of delicate micelle structures and their interaction with the surrounding solution. This can lead to a potential decrease in the recovery or content of compounds, possibly due to aggregation or degradation under prolonged centrifugation conditions.

#### 3.1.11. CPE Steps

The number of CPE steps significantly influences the success and quality of the extraction process, thereby impacting the final purity and yield of the extracted compounds [39]. Increasing the number of extraction steps is crucial for optimizing the overall effectiveness of this process, particularly in complex matrices where comprehensive recovery of target substances is challenging.

After the CPE Step 1, the TPC was 416.72 mg GAE/100 g dw, which constituted 75% of the overall phenolic content (Table 13). After a CPE Step 2, the TPC of 552.78 mg GAE/100 g dw was achieved. AA follows this trend as well. However, maximum recovery of carotenoids with a single CPE step was observed. In a study by Giovanoudis et al. [40], authors examined the impact of multiple CPE steps using Genapol X-080 at concentrations of 2%, 5%, and 10%, revealing that additional steps increased polyphenol recovery by approximately 35%. The most effective recovery was achieved with two extraction steps using 5% Genapol X-080. Additionally, Giovanoudis et al. [31] investigated ripe and unripe peaches using Tween 80, achieving polyphenol recovery rates of 54.24%, 70.92%, and 83.01% with concentrations of 2%, 5%, and 10% *w*/*v*, respectively, after one extraction step. The optimal approach involved two steps with 5% Tween 80, resulting in an 83% recovery rate. In contrast, Athanasiadis et al. [39] demonstrated that ultrasound-assisted CPE extraction of lemon peel with 20% *w*/*v* Span 20 achieved a 95% recovery rate in a single CPE step.

#### 3.1.12. Comparative Assessment of CPE, Water Extraction, Ultrasound, and Microwave Extraction

To assess the efficacy of the CPE method, a comparative analysis was conducted involving conventional water-based extraction and cutting-edge techniques such as microwave and ultrasonic extraction. Moreover, the influence of pretreatments such as microwave and ultrasonic extraction prior to CPE on enhancing the yield of polyphenols and carotenoids was examined. Detailed findings are presented in Table 14.

The highest yield of TPC and AA was noted for CPE, followed by microwave-assisted CPE, ultrasound-assisted CPE, microwave, ultrasound, and water extraction. The highest yield of TC order was CPE > Ultrasound-assisted CPE > Ultrasound extraction > Microwave-assisted CPE > Microwave extraction, while water extraction did not recover any carotenoids. This outcome was anticipated because carotenoids are known to dissolve in non-polar organic solvents rather than polar solvents like water. However, the CPE method employed water as the solvent, facilitated by surfactants such as Tween 80. This surfactant contains both polar and non-polar segments in its molecular structure, which allows it to bind carotenoids as non-polar substances to the non-polar part of the molecule. This mechanism enabled the incorporation of carotenoids into micelle, leading to their isolation from the plant material.

Further, microwave-assisted CPE emerges as another effective technique, with TPC reaching 427.77 mg GAE/100 g dw and AA at 974.79 μmol TE/100 g dw. In the study of Tang et al. [41], application of microwave-assisted cloud point extraction was optimized and compared to conventional extraction methods (heating reflux extraction and ultrasonic-assisted extraction). It revealed that not only higher recovery was obtained with microwave-assisted cloud point extraction, but also the flavonoids and alkaloids were successfully separated.

Ultrasound-assisted CPE significantly boosted the TPC to 345.56 mg GAE/100 g dw compared to ultrasound extraction alone (255.37 mg GAE/100 g dw). Additionally, ultrasound-assisted CPE demonstrated higher values for TC and AA compared to ultrasound extraction as well. Mai et al. [42] highlighted the efficacy of ultrasound-assisted extraction combined with CPE for enhancing flavonoid recovery and separation from *Euonymus alatus*. This method showed superior selectivity and efficiency compared to other extraction techniques such as pressurized microwave-assisted extraction (PMAE), ultrasonic-assisted extraction (UAE), heating reflux extraction (HRE), and cold maceration. Notably, UAE and PMAE achieved the highest recovery rates of catechin at 0.659 and 0.636 mg/g, respectively, surpassing cold maceration and HRE with catechin contents of 0.103 and 0.416 mg/g, respectively. However, the combination of UAE with CPE demonstrated the highest recovery rate of flavonoids, specifically catechin, at 0.684 mg/g. These findings underscore the effectiveness of integrating ultrasonic-assisted extraction with CPE.

#### 3.1.13. HPLC Phenols and Carotenoids Profiling

To facilitate a more detailed comparison, HPLC analysis was additionally used to identify and characterize the phenolic compounds and carotenoids in the selected horned melon peel extracts (Table 15).

Achieved total phenol content in horned melon peel extracts decreased in the following order: CPE (58.87 mg/100 g dw) > Water extraction (49.96 mg/100 g dw) > Ultrasound-assisted CPE (31.54 mg/100 g dw) > Microwave-assisted CPE (29.87 mg/100 g dw) > Microwave extraction (28.19 mg/100 g dw) > Ultrasound extraction (26.98 mg/100 g dw). Higher phenolic values obtained via the spectrophotometric method, compared to those measured by HPLC, could be due to interference from other substances in the extracts, such as ascorbic acid, sugars, and aromatic amines. These substances might affect the Folin–Ciocalteu assay, leading to an overestimation of the TPC [43]. Considering individual phenolic compounds, the highest yield in almost all extracts was observed for catechin. In CPE, catechin represents ~73% of total phenolics. Catechin as a polyphenolic flavonoid is notable for its antioxidant properties and potential health benefits. Catechin may support heart health, aid in weight management by boosting metabolism, and have cancer-preventive and antimicrobial effects. Additionally, incorporating catechin-rich foods into the diet can be a valuable strategy for promoting overall well-being [44]. Besides catechin, in the horned melon peel, extracts were identified, as well as p-hydroxybenzoic acid, gallic acid, protocatechin, caffeic acid, syringic acid, and vanillic acid. In the scientific literature, there is scarce information about the qualitative analysis of this type of fruit, and they mainly refer to the edible part. Thus, Vieira et al. [45] revealed the presence of high amounts of gallic acid and protocatechuic acid in horned melon fruit, while Barin et al. [46] showed a significant presence of benzoic acid, caffeic acid, and gallic acid in this fruit.

In the context of extracting phenolic compounds from horned melon peel, the use of surfactant water solutions proved to be more effective than pure water across all extraction techniques employed. This enhanced efficiency is particularly notable in the case of catechin, where surfactants play a significant role. However, an interesting observation was that gallic acid yields were higher in treatments that did not utilize surfactant-based CPE. This result can be attributed to the polarity of gallic acid. Gallic acid is inherently more polar than catechin due to its chemical structure, which includes a carboxyl group (–COOH) and multiple hydroxyl groups (–OH). These functional groups enhance gallic acid’s ability to form hydrogen bonds and interact with polar solvents, making it more soluble in water compared to less polar molecules. Consequently, gallic acid is effectively extracted from the peel even without the aid of surfactants. The surfactant-enhanced extraction method excels at extracting both polar and less polar compounds by improving their solubility in aqueous solutions. This approach not only increases the overall efficiency of the extraction process but also addresses environmental concerns.

The higher phenol content observed in the CPE and water extract can be attributed to the lower extraction temperature of 45 °C, compared to 60 °C for ultrasound and ultrasound-assisted CPE treatments, and the significant heat generated during microwave and microwave-assisted CPE. Phenolic compounds, being sensitive to heat, may degrade under higher temperatures, resulting in reduced content. Dorta et al. [47] noted that water extracts of mango by-products had lower phytochemical content at 75 °C compared to 50 °C. Similarly, Volf et al. [48] studied the effect of temperature on standard solutions of polyphenols such as catechin, gallic acid, and vanillic acid. They found that catechin, being particularly sensitive to heat, experienced a degradation rate of about 20% at 60 °C, which increased to 32% at 100 °C. This suggests that variations in extraction temperatures could lead to similar degradation patterns.

Carotenoids, another important class of phytochemicals found in horned melon peels, exhibit considerable variation in extraction efficiency depending on the method used. CPE yields the highest total carotenoid content (2 mg/100 g dw), followed by ultrasound-assisted CPE (1.82 mg/100 g dw), microwave-assisted CPE (1.44 mg/100 g dw), and ultrasound extraction (1.29 mg/100 g dw). Water-extraction and microwave-extraction methods yielded no detectable carotenoids. β-cryptoxhantin was the most abundant carotenoid extracts, followed by lutein and zeahanthin. β-carotene, notable for its non-polar structure, was effectively extracted using methods that included CPE, highlighting the techniques’ ability to handle both nonpolar substances due to the surfactant-enhanced solubility.

### 3.2. ANN Model

The significance of employing a backpropagation algorithm, a multilayer feed-forward ANN, which utilizes the adaptive gradient learning rule, as a crucial tool in biological information processing and data modeling. Such methods hold promise for the screening and optimization of various bioprocesses. In this investigation, the obtained optimal neural network model exhibited robust generalization capabilities concerning experimental data, enabling accurate predictions of the recovery of bioactive compounds with different polarities from the horned melon peel (Table 3). Utilizing 11 variables according to the experimental design, it was determined that the optimal number of neurons was seven (network MLP 14–7–3), yielding the highest r^2^ values during the training cycle for the three output variables (r^2^ reached 1.000 for all outputs). The training algorithm employed was BFGS 53, with identity activation functions for the hidden layer and output layer. The resultant ANN model for predicting the three output variables proved to be complex, with 129 weight biases, owing to the high nonlinearity inherent in the observed system. A high r^2^ value signifies that the variation was adequately accounted for, and the data fit the proposed model satisfactorily [49].

### 3.3. Global Sensitivity Analysis—Yoon’s Interpretation Method

**Figure 1:**
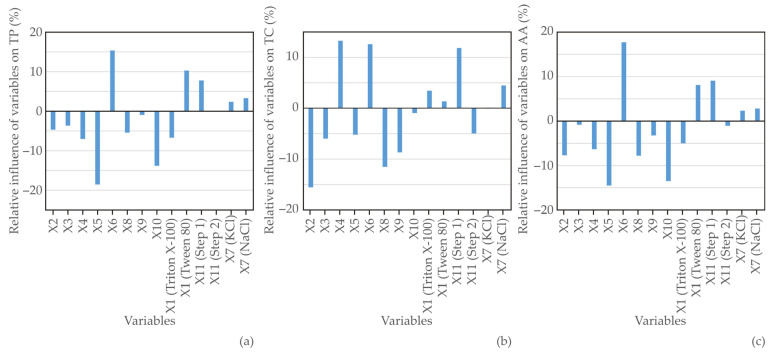
Relative influence of variables X1–X11 on (**a**) TP, (**b**) TC, and (**c**) AA. X1—surfactant type; X2—surfactant concentration; X3—solid:liquid ratio; X4—pH; X5—equilibration temperature; X6—equilibration time; X7—salt type; X8—salt concentration; X9—centrifugation speed; X10—centrifugation time; and X11—the number of CPE steps.

The impacts of various input variables (including surfactant type; surfactant concentration; solid:liquid ratio; pH value; equilibration temperature; equilibration time; salt type; salt concentration; centrifugation speed; centrifugation time; and the No. of CPE steps) on determining the most effective cloud point extraction process to enhance the recovery of bioactive compounds with different polarities from horned melon peel were investigated using the Plackett–Burman’s experimental design and Yoon’s interpretation method with a developed ANN model. The graphical representation of the results from the ANN model is depicted in Figure 1.

### 3.4. Multi-Objective Optimization of the Outputs of the ANN

The primary objective of this study was to optimize several output parameters: achieving the maximum TP, TC, and AA values. This optimization was conducted simultaneously for all constraints using an ANN model, with equal weight coefficients assigned to each variable. The constraints utilized in the optimization process were derived from the experimental dataset. The optimization task was performed using the ANN model through multi-objective optimization (MOO) calculations in Matlab, R2018b. The process involved 36 generations for the ANN model, with a population size set to 100 for each input variable, resulting in 22 points on the Pareto front.

Based on the calculated values of the tested parameters (refer to Table 2), it was determined that TP, TC, and AA reached the highest values of 348.56; 16.4, and 968.99, respectively. This result was obtained for the optimal sample, with parameter values: surfactant type = Tween 80, surfactant concentration = 2%; solid:liquid ratio = 1:100; pH = 6612; equilibration temperature = 35 °C; equilibration time = 60 min; salt type = NaCl; salt concentration = 16.4%; centrifugation speed = 7906× *g*; centrifugation time = 13.358 min; and No. of CPE steps = Step 1. It can be observed that the combination of ANN-based MOO optimization and Plackett–Burman experimental design has proven to be a powerful strategy for maximizing TC. A significantly higher content was observed for the optimal sample (~222% higher) compared to the optimal sample after OVAT screening. The notable enhancement in TC achieved using the Plackett–Burman design illustrates its capability to systematically and effectively determine optimal experimental conditions while considering complex interactions between variables that might be overlooked by the OVAT approach. This underscores the importance of utilizing advanced experimental designs and optimization methods to maximize biochemical yields.

Matsusaka and Kawabata [50] found that extracting horned melon peel with 50% aqueous ethanol (*v*/*v*) at 30 °C for 24 h resulted in a slightly higher polyphenol content (5 mg/g). However, comparing our phenolic compound yield with data from other studies is challenging due to variations in the specific horned melon cultivar and environmental conditions during its growth, which significantly influence the types and amounts of compounds present in the peel. Moreover, when comparing the optimal extraction time of 60 min achieved using CPE with the 24 h duration used by Matsusaka and Kawabata [41], the cost-effectiveness of the CPE approach becomes apparent. This method not only reduces extraction time but also offers environmental benefits (in terms of avoiding organic solvents) and cost savings, making it a favourable choice for obtaining phenolic compounds from horned melon peel. By significantly reducing solvent usage and operating at lower temperatures and shorter durations, CPE minimizes the environmental impact associated with chemical use and energy consumption, aligning with sustainable extraction practices.

Recent scientific literature shows a notable focus on closed-loop systems for reusing surfactants like Tween 80, particularly in the direct use of bioactive extracts obtained through CPE in diverse product applications. This interest is driven by the dual goals of sustainability and cost-effectiveness, along with the potential for technological advancements and broader applications in functional products across various industries. For instance, in yogurt production, which involves the use of emulsifiers like Tween 80, various technologies have been proposed to enhance them with plant extracts rich in polyphenols and antioxidants [51,52]. Almeida et al. [53] recommended utilizing Tween 80 for nanoencapsulation of curcumin, a polyphenol, to be used as a dietary supplement in yogurt. Another approach involves using plant extracts prepared in surfactant solutions to create nanoemulsions that encapsulate polyphenols and other biologically active compounds [54,55]. Several published studies have highlighted the benefits of such colloidal systems in drug delivery [47,48]. Nevertheless, to effectively incorporate the extracts obtained using the proposed method in this study into industrial applications, further research is essential. Specifically, thorough investigations are needed into the safety profile, microbiological aspects, and stability of the final products.

### 3.5. Experimental Verification of ANN Model Results in Laboratory Testing

In order to evaluate the accuracy of the developed ANN models, experimental verification was conducted in the laboratory. The verification scheme involved testing TP, TC, and AA values for four modified samples from Table 2 (PB design). These samples had altered solid–liquid ratio values and modified equilibrium temperatures. Specifically, the solid–liquid ratio for Samples 9 and 10 (Table 2) was adjusted to 0.02. The equilibrium temperature for Sample 3 was set to 35 °C, while for Sample 6, it was set to 55 °C. The input parameters used for the CPE process are presented in Table 15. Additionally, the optimization results of the ANN model were examined by experimentally validating the TP, TC, and AA values obtained through multi-objective optimization, as detailed in Section 3.3. The outcomes of these experimental procedures are also presented in Table 16.

The ANN model was used to calculate the TP, TC, and AA values for the modified samples (3, 6, 9, and 10, in Table 15), while the optimal sample’s TP, TC, and AA values were previously calculated and presented in Section 3.3. The experimental values for TP, TC, and AA are also presented in Table 15.

The results demonstrate good agreement between the model predictions and experimental values across all samples. Minor discrepancies were observed, as expected in practical applications. The highest deviations were noted in TP for Sample 6 (relative error of −3.54%) and TC for Sample 10 (relative error of −4.72%), both within acceptable ranges. These findings suggest that the ANN model is reliable and performs well in predicting the TP, TC, and AA parameters.

## 4. Conclusions

The present study focused on optimizing the CPE process for the recovery of polyphenols and carotenoids from horned melon peel through a combination of one-variable-at-a-time (OVAT) experimentation, Plackett–Burman design, and Artificial Neural Network (ANN) analysis. This comprehensive approach facilitated a detailed understanding of the factors influencing CPE and enabled the identification of optimal extraction conditions. Under the identified optimal conditions—using Tween 80 as the surfactant at a concentration of 2%, a solid-to-liquid ratio of 1:100, pH 6.612, equilibration at 35 °C for 60 min, NaCl as the salt with a concentration of 16.4%, centrifugation at 7906× *g* for 13.358 min, and a single CPE step—the highest yields of total phenolics (352.49 mg GAE/100 g), total carotenoids (16.59 mg β-carotene/100 g), and antioxidant activity (989.02 μmol TE/100 g) were achieved. HPLC profiling revealed the presence of catechin, p-hydroxybenzoic acid, gallic acid, protocatechin, caffeic acid, syringic acid, and vanillic acid as phenols, as well as β-cryptoxhantin, lutein, zeahanthin, and β-carotene as carotenoids. The findings demonstrate that CPE not only outperforms traditional extraction methods but also reveal new opportunities for the development of the highly efficient process of green extraction of both hydrophilic and hydrophobic compounds, allowing the extraction of bioactive compounds from complex matrices, such as different by-products. This offers promising opportunities for the integration of obtained valuable compounds from by-products into the food industry, as well as excellent waste management solutions. Future research should focus on the broader applications of the CPE extracts, including their integration into functional products and evaluation of their safety, microbiological aspects, and stability. Additionally, exploring closed-loop systems for surfactant reuse and advancements in colloidal systems for encapsulating bioactive compounds will be crucial for expanding the practical applications of these findings.

## Figures and Tables

**Table 1:** CPE parameters for extraction of bioactive compounds from horned melon peel.

No.	Parameters	Variables	Units
1.	Surfactant type	Triton X-100; Tween 80; Span 85; Ceteareth-12	-
2.	Surfactant concentration	2; 4; 6; 8; 10	%, *w*/*v*
3.	Solid:liquid ratio	1:10; 1:30; 1:50; 1:70; 1:100	*w*/*v*
4.	pH	1.5; 3.0; 4.5; 6.0; 7.5	-
5.	Equilibration temperature	35; 45; 55; 65; 75	°C
6.	Equilibration time	20; 30; 40; 50; 60	Minutes
7.	Salt type	NaCl; KCl; CaCl_2_	%, *w*/*v*
8.	Salt concentration	6; 10; 14; 16; 18	-
9.	Centrifugation speed	2473; 5564; 9892	*g*
10.	Centrifugation time	10; 15; 20	Minutes
11.	CPE steps	1; 2	-

**Table 2:** Plackett–Burman experimental design (PBD) with obtained responses.

Pattern	X1	X2	X3	X4	X5	X6	X7	X8	X9	X10	X11	TPC (mg GAE/100 g dw)	TC (mg β-Carotene/100 g dw)	AA (μmol TE/100 g dw)
1	Tween 80	10	1:10	7.5	75	60	NaCl	18	9892	20	Step 2	0	0	0
2	Triton X-100	10	1:100	7.5	75	60	KCl	16	2473	20	Step 1	0	12.58	164.88
3	Triton X-100	2	1:10	1.5	75	60	NaCl	16	2473	10	Step 2	105.61	7.89	493.27
4	Tween 80	2	1:100	7.5	35	60	NaCl	18	2473	10	Step 1	348.56	16.4	968.99
5	Triton X-100	10	1:100	1.5	75	20	NaCl	18	9892	10	Step 1	0	0	0
6	Triton X-100	2	1:10	1.5	35	60	KCl	18	9892	20	Step 1	142.11	6.81	487.2
7	Triton X-100	2	1:100	7.5	35	20	NaCl	16	9892	20	Step 2	0	8.57	0
8	Tween 80	2	1:100	1.5	75	20	KCl	18	2473	20	Step 2	0	0	0
9	Tween 80	10	1:100	1.5	35	60	KCl	16	9892	10	Step 2	335.48	1.86	825.93
10	Tween 80	10	1:10	1.5	35	20	NaCl	16	2473	20	Step 1	172.22	5.05	471.32
11	Triton X-100	10	1:10	7.5	35	20	KCl	18	2473	10	Step 2	0	0	0
12	Tween 80	2	1:10	7.5	75	20	KCl	16	9892	10	Step 1	109.33	8.61	407.35

Variables: X1—surfactant type; X2—surfactant concentration (%, *w*/*v*); X3—solid:liquid ratio (*w*/*v*); X4—pH; X5—equilibration temperature (°C); X6—equilibration time (min); X7—salt type; X8—salt concentration (%, *w*/*v*); X9—centrifugation speed (*g*); X10—centrifugation time (min); X11—CPE steps.

**Table 3:** Evaluation of different types of surfactants.

Run Number	Type of Surfactant	TPC (mg GAE/100 g dw)	TC (mg β-Carotene/100 g dw)	AA (μmol TE/100 g dw)
1.	Tween 80	81.72 ± 0.43 ^b^	6.27 ± 0.63 ^b^	226.47 ± 13.59 ^b^
2.	Triton X-100	20.93 ± 0.43 ^a^	5.81 ± 0.17 ^a^	140.14 ± 11.76 ^a^
3. *	Ceteareth-12	-	-	-
4. *	Span 85	-	-	-

Means in the same column with different superscripts are statistically different (*p* < 0.05). * No phase separation detected.

**Table 4:** Evaluation of different surfactant concentrations.

Run Number	Concentration of Surfactant (%)	TPC (mg GAE/100 g dw)	TC (mg β-Carotene/100 g dw)	AA (μmol TE/100 g dw)
1.	2	62.59 ± 0.48 ^a^	6.22 ± 0.46 ^a^	204.85 ± 0.32 ^a^
2.	4	104.19 ± 0.43 ^b^	10.68 ± 0.10 ^c^	302.50 ± 0.19 ^b^
3.	6	107.16 ± 1.79 ^c^	10.70 ± 0.13 ^c^	288.17 ± 22.02 ^b^
4.	8	145.65 ± 2.48 ^d^	10.34 ± 0.02 ^b^	352.06 ± 22.80 ^c^
5.	10	150.97 ± 1.64 ^e^	10.30 ± 0.31 ^b^	409.14 ± 16.09 ^d^

Means in the same column with different superscripts are statistically different (*p* < 0.05).

**Table 5:** Evaluation of solid-to-liquid ratio.

Run Number	Solid:Liquid Ratio	TPC (mg GAE/100 g dw)	TC (mg β-Carotene/100 g dw)	AA (μmol TE/100 g dw)
1.	1:10	107.19 ± 2.14 ^a^	5.32 ± 0.44 ^a^	320.65 ± 8.94 ^a^
2.	1:30	134.42 ± 2.08 ^c^	8.79 ± 0.34 ^b^	479.08 ± 91.21 ^b^
3.	1:50	201.35 ± 2.68 ^e^	10.75 ± 0.32 ^c^	617.73 ± 84.63 ^c^
4.	1:70	179.37 ± 1.55 ^d^	10.25 ± 0.43 ^c^	605.51 ± 2.72 ^c^
5.	1:100	122.01 ± 0.30 ^b^	nd *	320.85 ± 9.66 ^a^

Means in the same column with different superscripts are statistically different (*p* < 0.05). * Not detected.

**Table 6:** Evaluation of pH value.

Run Number	pH	TPC (mg GAE/100 g dw)	TC (mg β-Carotene/100 g dw)	AA (μmol TE/100 g dw)
1.	1.5	163.50 ± 1.45 ^d^	3.07 ± 0.16 ^a^	403.37 ± 1.79 ^c^
2.	3.0	198.86 ± 1.45 ^e^	5.50 ± 0.23 ^b^	622.42 ± 5.21 ^d^
3.	4.5	125.88 ± 1.46 ^c^	10.77 ± 1.40 ^d^	313.05 ± 23.46 ^a^
4.	6.0	77.73 ± 1.95 ^a^	10.03 ± 1.42 ^d^	350.02 ± 50.64 ^b^
5.	7.5	83.25 ± 1.28 ^b^	8.29 ± 0.32 ^c^	404.07 ± 56.85 ^c^

Means in the same column with different superscripts are statistically different (*p* < 0.05).

**Table 7:** Evaluation of equilibration temperature.

Run Number	Temperature (°C)	TPC (mg GAE/100 g dw)	TC (mg β-Carotene/100 g dw)	AA (μmol TE/100 g dw)
1.	35	270.04 ± 7.76 ^d^	2.24 ± 0.03 ^a^	798.89 ± 16.75 ^d^
2.	45	279.81 ± 7.08 ^d^	2.70 ± 0.27 ^b^	1085.39 ± 2.24 ^e^
3.	55	194.62 ± 7.62 ^c^	2.55 ± 0.18 ^ab^	795.08 ± 2.80 ^d^
4.	65	174.98 ± 4.14 ^b^	2.58 ± 0.31 ^ab^	725.26 ± 12.78 ^c^
5.	75	142.34 ± 1.63 ^a^	2.18 ± 0.45 ^a^	428.97 ± 9.59 ^a^

Means in the same column with different superscripts are statistically different (*p* < 0.05).

**Table 8:** Evaluation of equilibration time.

Run Number	Time (min)	TPC (mg GAE/100 g dw)	TC (mg β-Carotene/100 g dw)	AA (μmol TE/100 g dw)
1.	20	293.71 ± 4.85 ^e^	3.29 ± 0.30 ^c^	1091.23 ± 24.96 ^c^
2.	30	279.81 ± 7.08 ^d^	2.45 ± 1.21 ^b^	1014.61 ± 8.20 ^ab^
3.	40	194.62 ± 7.62 ^c^	1.88 ± 0.69 ^a^	1025.64 ± 14.26 ^b^
4.	50	174.98 ± 4.14 ^b^	2.38 ± 1.74 ^b^	1001.26 ± 30.23 ^a^
5.	60	142.34 ± 1.63 ^a^	2.30 ± 0.23 ^b^	1008.52 ± 8.51 ^a^

Means in the same column with different superscripts are statistically different (*p* < 0.05).

**Table 9:** Evaluation of salt type.

Run Number	Type of Salt	TPC (mg GAE/100 g dw)	TC (mg β-Carotene/100 g dw)	AA (μmol TE/100 g dw)
1.	NaCl	295.92 ± 2.67	3.59 ± 0.74	1095.84 ± 10.97
2. *	KCl	/	/	/
3. *	CaCl_2_	/	/	/

* No phase separation detected.

**Table 10:** Evaluation of salt concentration.

Run Number	Salt Concentration (%)	TPC (mg GAE/100 g dw)	TC (mg β-Carotene/100 g dw)	AA (μmol TE/100 g dw)
1. *	10	/	/	/
2. *	14	/	/	/
3.	16	236.49 ± 3.21 ^b^	6.65 ± 0.43 ^b^	867.23 ± 49.91 ^b^
4.	18	165.93 ± 4.28 ^a^	5.44 ± 0.49 ^a^	480.02 ± 41.52 ^a^

Means in the same column with different superscripts are statistically different (*p* < 0.05). * No phase separation detected.

**Table 11:** Evaluation of centrifugation speed.

Run Number	Centrifugation Speed (g)	TPC (mg GAE/100 g dw)	TC (mg β-Carotene/100 g dw)	AA (μmol TE/100 g dw)
1.	2473	296.67 ± 5.62 ^c^	7.02 ± 0.35 ^c^	740.32 ± 76.10 ^b^
2.	5564	275.82 ± 6.03 ^b^	6.26 ± 0.41 ^b^	731.40 ± 47.02 ^b^
3.	9892	260.77 ± 4.95 ^a^	5.56 ± 0.31 ^a^	667.34 ± 22.28 ^a^

Means in the same column with different superscripts are statistically different (*p* < 0.05).

**Table 12:** Evaluation of centrifugation time.

Run Number	Centrifugation Time (min)	TPC (mg GAE/100 g dw)	TC (mg β-Carotene/100 g dw)	AA (μmol TE/100 g dw)
1.	10	390.74 ± 0.85 ^c^	5.01 ± 0.44 ^b^	1115.92 ± 7.49 ^c^
2.	15	192.93 ± 1.43 ^b^	3.98 ± 0.79 ^a^	869.54 ± 4.61 ^b^
3.	20	187.29 ± 3.84 ^a^	4.96 ± 0.37 ^b^	790.96 ± 10.15 ^a^

Means in the same column with different superscripts are statistically different (*p* < 0.05).

**Table 13:** Evaluation of the No. of CPE steps.

Run Number	Steps	TPC (mg GAE/100 g dw)	TC (mg β-Carotene/100 g dw)	AA (μmol TE/100 g dw)
1.	Step 1	416.72 ± 4.33 ^b^	4.57 ± 0.23 ^a^	998.64 ± 2.09 ^b^
2.	Step 2	136.06 ± 1.19 ^a^	nd *	362.82 ± 4.63 ^a^
	Sum	552.78 ± 5.52	4.57 ± 0.23	1361.46 ± 6.72

Means in the same column with different superscripts are statistically different (*p* < 0.05). * Not detected.

**Table 14:** Evaluation of CPE, water extraction, ultrasound, ultrasound-assisted CPE, microwave, and microwave-assisted CPE.

Run Number	Treatment	TPC (mg GAE/100 g dw)	TC (mg β-Carotene/100 g dw)	AA (μmol TE/100 g dw)
1.	CPE *	417.89 ± 2.41 ^f^	4.98 ± 0.08 ^e^	1000.53 ± 5.48 ^f^
2.	Water extraction	211.43 ± 3.22 ^a^	nd **	437.28 ± 10.22 ^a^
3.	Ultrasound extraction	255.37 ± 1.83 ^b^	2.68 ± 0.37 ^c^	865.63 ± 13.80 ^c^
4.	Ultrasound-assisted CPE	345.56 ± 1.68 ^c^	3.96 ± 0.06 ^d^	956.82 ± 0.62 ^d^
5.	Microwave extraction	338.97 ± 8.65 ^d^	0.22 ± 0.07 ^a^	745.05 ± 32.41 ^b^
6.	Microwave-assisted CPE	407.77 ± 1.57 ^e^	1.97 ± 0.11 ^b^	974.79 ± 2.35 ^e^

Means in the same column with different superscripts are statistically different (*p* < 0.05). * Single step; ** not detected.

**Table 15:** HPLC phenols and carotenoids profiling of horned melon peel extracts.

	CPE	Water Extraction	Ultrasound Extraction	Ultrasound-Assisted CPE	Microwave Extraction	Microwave-Assisted CPE
Phenols (mg/100 g dw)						
p-Hydroxybenzoic acid	0.41 ± 0.00 ^a^	0.67 ± 0.00 ^b^	0.88 ± 0.01 ^d^	0.71 ± 0.00 ^c^	1.40 ± 0.03 ^e^	nd *
Gallic acid	3.75 ± 0.01 ^a^	9.64 ± 0.02 ^c^	14.23 ± 1.25 ^d^	3.72 ± 0.02 ^a^	7.62 ± 0.10 ^b^	3.57 ± 0.01 ^a^
Protocatechin	2.90 ± 0.02 ^b^	3.32 ± 0.01 ^c^	2.75 ± 0.01 ^a^	2.94 ± 0.01 ^b^	3.39 ± 0.02 ^c^	3.49 ± 0.00 ^c^
Caffeic acid	0.55 ± 0.01 ^e^	0.38 ± 0.00 ^b^	0.34 ± 0.00 ^a^	0.46 ± 0.00 ^d^	0.41 ± 0.01 ^c^	0.69 ± 0.00 ^f^
Catechin	42.86 ± 1.17 ^f^	29.07 ± 0.01 ^e^	3.27 ± 0.04 ^a^	15.58 ± 1.02 ^d^	8.37 ± 0.06 ^b^	13.02 ± 0.05 c
Syringic acid	2.61 ± 0.00 ^d^	1.75 ± 0.00 ^b^	1.55 ± 0.00 ^a^	2.13 ± 0.01 ^c^	1.82 ± 0.01 ^b^	2.49 ± 0.00 ^d^
Vanillic acid	5.79 ± 0.01 ^c^	5.14 ± 0.03 ^b^	3.96 ± 0.02 ^a^	6.00 ± 0.00 ^d^	5.18 ± 0.21 ^b^	6.60 ± 0.03 ^e^
Total phenols	58.87 ± 1.22 ^e^	49.96 ± 0.07 ^d^	26.98 ± 1.33 ^a^	31.54 ± 1.06 ^c^	28.19 ± 0.44 ^a,b^	29.87 ± 0.09 ^b^
Carotenoids (mg/100 g dw)						
β-cryptoxanthin	1.91 ± 0.01 ^d^	nd *	1.27 ± 0.01 ^a^	1.77 ± 0.02 ^c^	nd	1.37 ± 0.01 ^b^
Lutein	0.04 ± 0.00 ^c^	nd *	0.01 ± 0.00 ^a^	0.03 ± 0.00 ^b^	nd	0.04 ± 0.00 ^c^
Zeaxanthin	0.03 ± 0.00 ^c^	nd *	0.01 ± 0.00 ^a^	0.01 ± 0.00 ^a^	nd	0.02 ± 0.00 ^b^
β-carotene	0.02 ± 0.00 ^b^	nd *	nd *	0.01 ± 0.00 ^a^	nd	0.01 ± 0.00 ^a^
Total carotenoids	2.00 ± 0.01 ^d^	nd *	1.29 ± 0.01 ^a^	1.82 ± 0.02 ^c^	nd	1.44 ± 0.01 ^b^

Means in the same column with different superscripts are statistically different (*p* < 0.05). * Not detected.

**Table 16:** Experimental verification of ANN model.

		Modified Samples for Verification of ANN Model				
	Parameter	3	6	9	10	Optimal Sample
	X1	Triton X-100	Triton X-100	Tween 80	Tween 80	Tween 80
	X2	2	2	10	10	2
	X3	1:10	1:10	1:50	1:50	1:100
	X4	1.5	1.5	1.5	1.5	6.61
	X5	35	55	35	35	35
	X6	60	60	60	20	60
	X7	NaCl	KCl	KCl	NaCl	NaCl
	X8	16	18	16	16	16
	X9	2473	9892	9892	2473	7906
	X10	10	20	10	20	13.36
	X11	Step 2	Step 1	Step 2	Step 1	Step 1
ANN model	TP	107.48	139.12	346.43	165.95	352.49
values	TC	8.01	7.00	1.81	5.11	16.59
	AA	468.75	465.77	812.89	483.30	989.02
Experimental	TP	105.42	144.23	356.28	165.49	341.55
values	TC	8.37	7.02	1.82	5.37	16.02
	AA	459.51	465.22	807.12	495.19	983.02

Variables: X1—surfactant type; X2—surfactant concentration (%, *w*/*v*); X3—solid:liquid ratio (*w*/*v*); X4—pH; X5—equilibration temperature (°C); X6—equilibration time (min); X7—salt type; X8—salt concentration (%, *w*/*v*); X9—centrifugation speed (g); X10—centrifugation time (min); X11—CPE steps.

## Data Availability

The original contributions presented in the study are included in the article, further inquiries can be directed to the corresponding author.
